# Unveiling the lockdown effects: exploring behavior, dietary habits and weight changes in rural Egypt during COVID-19 lockdown: a cross-sectional retrospective study

**DOI:** 10.1186/s41043-024-00558-8

**Published:** 2024-06-15

**Authors:** Mahmoud Reda Saleh, Mohamed Y. Abdelgaied, Naira Galal, Mai Tarek, Aya Fouda, Khaled Abdelkawy

**Affiliations:** 1 Faculty of Pharmacy, Kafrelsheikh University, Kafr- Elsheikh, 33511 Egypt; 2 Faculty of Pharmacy and Pharmaceutical Sciences, University of Alberta, Edmonton, AB T6G 2E1 Canada

**Keywords:** COVID-19, Dietary habits, Lockdown, Obesity, Egypt

## Abstract

**Background:**

The COVID-19 lockdown significantly impacted dietary habits and body weights globally, particularly in Egypt, where 57.03% of the population resides in rural areas, despite lack of information. The study examines the impact of COVID-19 lockdown on the weight changes of the rural Egyptian population through behavioral, physical, and dietary changes.

**Methods:**

A cross-sectional online survey using Microsoft Forms was distributed in Delta regions in Egypt. The questionnaire used a modified version of the validated 14- items PREDIMED MedDiet Adherence Screener (MEDAS). The first part of the questionnaire addressed sociodemographic variables whereas the second one included questions related to dietary, behavioral and weight changes of participants. These changes were statistically tested for significance in relation to BMI, gender, home living, current job and family history of obesity.

**Results:**

A total of 306 participated in the study (70% females, 13% obese, 95% living with family, 56% university students, and 36% with family history of obesity). Obese showed a significant increase in sweet intake whereas underweight and normal weight people displayed a significant decrease in eating desire. Both females and males showed significant increase in consumption of fruits and vegetables with significant decrease in soft drink. However, women showed a significant decrease in sport activity relative to men. Participants living with family showed an increase in sweet intake while those living alone explored an increase in meal frequency. Employers revealed a significant decrease in sport activities and people with family history of obesity reported more sleeping times than those without family history of obesity.

**Conclusion:**

During Covid-19 quarantine, Egyptians’ eating habits improved, but daily routines were disrupted. Raising awareness about obesity and providing guidance on maintaining activity, energy, and mood is crucial for future quarantine situations.

## Introduction

On January 30, 2020, the World Health Organization (WHO) designated the COVID-19 a worldwide health emergency [1]. Several governments have had to make difficult choices as a result of this epidemic, beginning with the rigorous lockdown of the majority of social and economic activity, the imposition of curfews, and the prohibition of any gatherings or parties in groups. Universities, secondary schools, and other indoor institutions were closed, and the majority of workers were asked to work from home [2]. The reactions of various populations to the COVID-19 lockdown showed significant variation. Above all, Egypt requires special attention because, on February 14, 2020, it was the first nation to report a COVID-19 instance involving a Chinese tourist [3]. Being the most populated country in the Arab world, Egypt has a dense population that is expanding quickly, which has increased concerns about rapid virus transmission. Egyptians are renowned for their fondness of socializing over food and dining in public [4].

People’s basic way of life was abruptly altered, societal structures were upset, and uncertainty about the future created a sense of despondency [5]. In actuality, while some societies experienced growing social isolation, others benefited from a well-organized social system supported by families. In addition, people were compelled to spend far longer hours at home than normal; this gave them more time for cooking and snacking in addition to increasing their emotional eating, which was exacerbated by the stress and worry of the situation [4]. In addition, participants focused more on foods that boost immunity, like citrus fruits, ginger, almonds, and turmeric, in the hopes of protecting themselves against the COVID-19 effects [6]. People kept non-perishable goods like canned food and dried beans in storage because they were dubious about the availability of food and other resources. The growth of online grocery shopping has made it possible for consumers to more thoroughly assess the nutritional content and quality of food than in the past [7]. With the disruption of international trade between nations, people’s interest in domestic products increased. Furthermore, the majority of people’s physical activity was restricted by this overeating and the closed, out-of-door clubs and gyms, which led to an energy imbalance—a high energy intake and a low energy expenditure [4]. Their eating habits, level of physical activity, and state of mind all changed, and this had an impact on their body weight. More research is needed to fully understand these developments in Egypt, as there is currently little data available.

This study used a modified validated questionnaire that was distributed online across various social platforms among Egyptian governorates in order to assess the behavioral, physical, and dietary changes made by the rural Egyptian population “57.03% of the population population”, during the COVID-19 lockdown and to ascertain their effect on changes in weight [8]. By examining these variables, the Egyptian government can more effectively develop their suggestions in the event that a lockdown occurs in the future.

## Methods

### Study design and participants

Six of Egypt’s most populous cities were the focus of a cross-sectional study on the country’s rural population. The study was conducted between April and August 2020, during the lockdown period. The participants completed a voluntary, anonymous questionnaire, were told of the aim of the study, and were asked if they could use and publish the information gathered. Participants in the study had to be adults 18 years of age or older; those who were younger or had concomitant diseases such thyroid, diabetes, ascites, active hepatic disease, or renal abnormalities were not allowed to participate. By using strict selection criteria, it was made sure that any alterations that were noticed were solely related to the COVID-19 lockdown’s impacts.

### Procedure

An online questionnaire was used, leveraging the user-friendly Microsoft Forms platform, in order to optimize the population’s reach and inclusion. Widespread distribution of the questionnaire was achieved by a number of means, including well-known social media sites like Facebook and Twitter, professional networking sites like LinkedIn, and instant messaging apps like WhatsApp. A snowball sampling technique was used to carry out this distribution plan, allowing participants to distribute the questionnaire across their networks and connections. The online questionnaire was completely voluntary and we made it clear for the respondents that they can reject to receive survey invitations before starting response to questionnaire.

### Questionnaire design

The survey consisted of 41 closed-ended, multiple-choice items divided into two portions, which are shown in Supplementary Table S1. The first part of the study collected data related to sociodemographic variables, including age, gender, body mass index (BMI), place of residence, type of housing, Presence of children, employment status, and educational attainment. The second section consists of 28 questions that particularly address eating patterns, such as the quantity and variety of foods, food and drink consumption rates, and behavioral changes, such as starting an exercise regimen in addition to changing weight. The second part of the questionnaire was a modification of the validated 14-items PREDIMED Mediterranean diet (MedDiet) Adherence Screener (MEDAS-14) [9]. Due to religious convictions, alcohol consumption was not included in the study because it was an uncommon occurrence in Egypt [10]. The original MEDAS-14 questionnaire is in English language. However, we made an Arabic translation of the questionnaire to be easily understood by the participants. The added questions to reflect changes in physical activity and behavior were obtained from previous questionnaires in literatures or from experts or professors in behavior and public health at University of Kafrelsheikh [4]. The survey employed a closed-ended, multiple-choice format that give specific answer to the question to facilitate a statistical analysis, like the following example “Consumption of fruit and vegetable” the available answers were 1-normal, 2-more, 3-less” and the participants had to choose only one option. To ensure all questions were answered to get complete questionnaire application, each question has red asterisk (*) to indicate that the question had to be answered before submission unless error message will appear and the questionnaire application would not be submitted. By this way, we ensured the completeness of our questionnaire before online submission. As the questionnaire was conducted using Google Form and to prevent duplicate answers, we limited responses by Google account. Users responding to the form had to sign in with a Google account to respond, with each account limited to a single response. Google forms have many advantages like easy to use, simple and feasible for all participants. Before beginning the questionnaire, participants were informed about the purpose of the study and requested permission to use and publish the data from the study. The survey did not have any questions that might reveal respondent’s identity. In order to ensure the validity and reliability of participants´ response, pilot study of 25 participants who answered the survey twice, separated by five weeks, was carried out to verify the internal validity (reliability) of the questions. With Cronbach’s alpha, the survey’s reliability was evaluated. In more details, the implemented pilot study showed good internal validity and reliability with Cronbach’s alpha = 0.85. The Clinical Pharmacy Research Center at Kafrelsheikh University’s Faculty of Pharmacy supervised the coordination of the Egyptian study.

### Statistical analysis

The statistical analysis was conducted using version 26 of the Statistical Package for Social Sciences (SPSS) software. A statistical analysis was conducted to determine the significance of the dietary, behavioral, and weight changes with respect to age, gender, body mass index (BMI), residential location, living arrangements, and education level. Numbers and percentages were used to represent categorical variables. The Kolmogorov-Smirnov and Shapiro-Wilk statistical tests were used to first determine if continuous variables were normally distributed. Data that were not normally distributed were described by the median (interquartile range), whereas normally distributed data were described by the mean (standard deviation). To compare groups for categorical data, a chi-square or Fisher’s exact test was employed. Unless the Mann-Whitney U test was utilized, an unpaired Student t-test was performed for continuous data with normally distributed data. Cronbach’s alpha was used to test the reliability (internal consistency) of the scales of the questionnaire. Tests with *P* value < 0.05 were identified as statistically significant.

## Results

### Demographic characteristics of participants

**Fig. 1:**
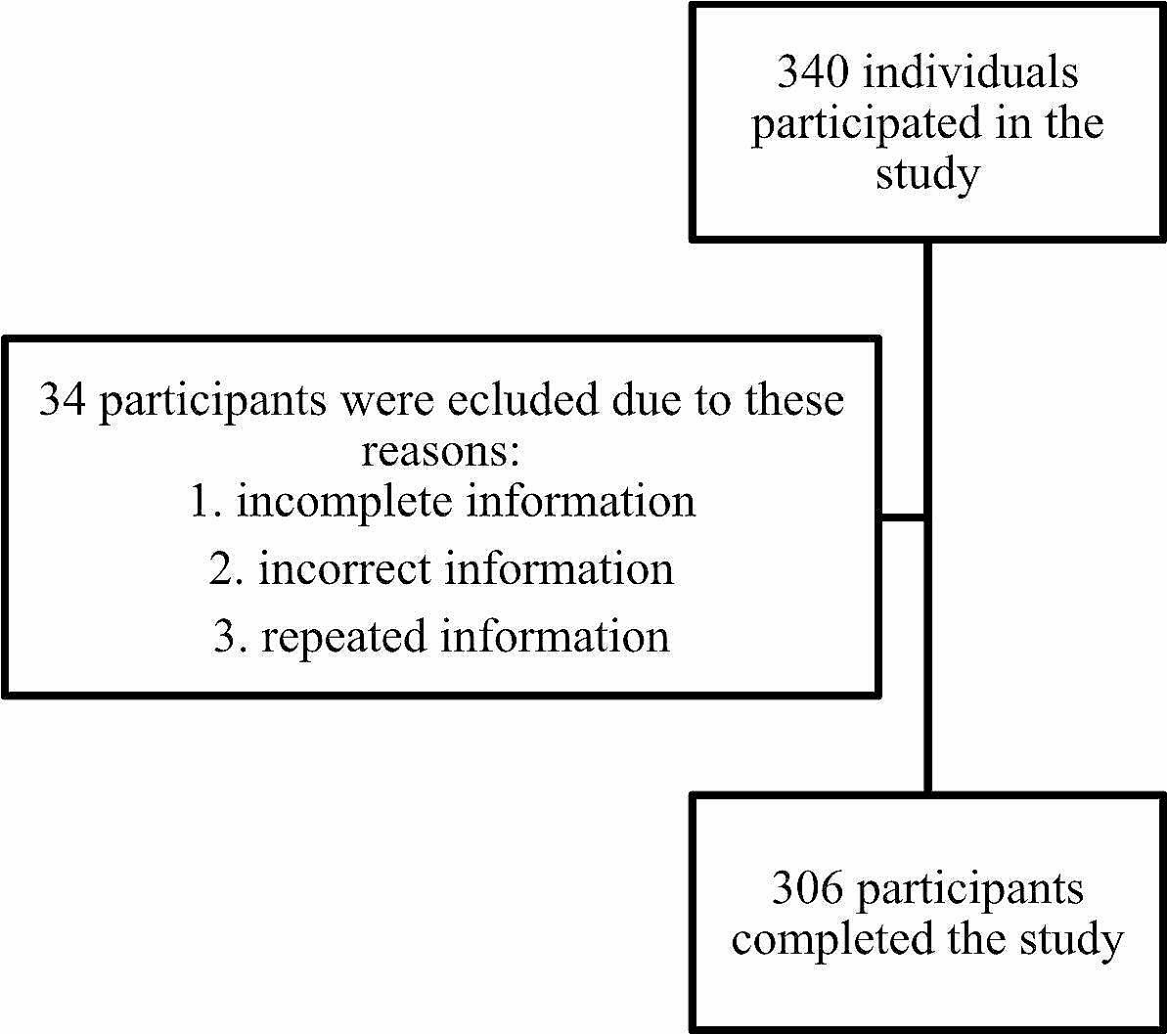
Flow chart of patients’ enrollment in the study

Although 340 individuals participated in the research, only 306 finished the questionnaire. As shown in Fig. 1, a total of 34 participants were eliminated from the study due to information misrepresentation or incomplete information such as living outside of Egypt. The majority of participants (70%) were female, and 83% of them were between the ages of 20 and 35. This study included the densely populated Egyptian rural cities, with Kafrelsheikh serving as the primary participant city. Furthermore, according to BMI, 46% of people had a normal body weight, whereas 51% of people were overweight or obese. In addition, 95% of participants reside with their families, demonstrating the strong social bonds among Egyptians. The majority of participants have advanced degrees because they are better at using surveys. 64% of the participants in total said they had no family history of obesity as shown in Table 1.

**Table 1:** Demographic and anthropometric characteristics of questionnaire respondents during the COVID-19 lockdown in Egypt

Baseline characteristics	Frequency, n (%)
**Age (years)**	
20–35	254 (83%)
35–50	9 (2.9%)
< 20	39 (13%)
> 50	4 (1.3%)
**Gender**	
Male	92 (30%)
Female	214 (70%)
**BMI**	
Under weight	9 (2.9%)
Normal	142 (46.4%)
Overweight	114 (37.25%)
Obese	41 (13.4%)
**Governorate**	
Behera	8 (2.6%)
Qalubia	11 (3.59%)
Gharbia	19 (6.2%)
Kafrelsheikh	222 (72.55%)
Menoufia	14 (4.57%)
Dakahlia	19 (6.2%)
Sharqia	13 (4.2%)
**Place of Residence**	
Alone or sharing friends	15 (4.9%)
With family	291 (95%)
**Children in Care**	
No	232 (76%)
Yes	74 (24%)
**Work Status**	
Employed	91 (30%)
Unemployed	41 (13%)
Retired	2 (0.7%)
Student	172 (56%)
**Education Level**	
Postgraduate degrees “Diploma, MSc, PhD”	52 (17%)
Secondary or less	24 (7.8%)
Bachelor	230 (75%)
**Family history of obesity**	
No	197 (64%)
Yes	109 (36%)

BMI: Body mass index

### General findings

As shown in Tables 2 and 16% of those who were evaluated had lost weight during the lockdown, while 34% had gained it. Of these weight changes, about 40% were planned. Out of the total, 36% of people said they didn’t exercise during lockdown, and 30% reported their exercise rate dropped. Conversely, 10% of participants increased the intensity of their workout. Precisely, 50% of the subjects revealed sleeping more than they had previously, while 38% reported no change at all. In addition, 64% reported experiencing behavioral changes, including sadness or disinterest. Of the total, 68.3% reported maintaining their normal daily meals. The intake of fruits and vegetables increased in 41.5% of the participants and remained normal within 51.3% of them. The same way, 72.5% maintained their normal consumption levels of protein. Furthermore, 23.5% of participants reported a rise in their consumption of sweets. 27.8% declared to have an enhanced appetite, while 53.9% had no changes.

**Table 2:** Participants’ dietary, behavioral and weight changes in relation to body mass index and gender

Questions	Response	Total	BMI Thin (*n* = 9) Normal (*n* = 142) Overweight(*n* = 114) Obese(*n* = 41)	*P*	Gender Male ( *n* = 92 ) Female (*n* = 214 )	*P*
**Weight change during covid-19**	Normal Increase Decrease	153 (50.0%) 104(34.0%) 49(16.0%)	6(66.7%) 93(65.5%) 44(38.6%) 10(24.4%) 1(11.11%) 26(18.3%) 49(43.0%) 28(68.3%) 2(22.22%) 23(16.2%) 21(18.4%) 3(7.3%)	< 0.001*	49 (53.3%) 104(48.60%) 32(34.8%) 72(33.6%) 11(12.0%) 38(17.8%)	0.437
**Weight change is intended**	Yes No	123(40.2%) 183(59.8%)	3(33.33%) 49(34.5%) 51(44.7%) 20(48.8%) 6(66.67%) 93(65.5%) 63(55.3%) 21(51.2%)	0.229	37(40.22%) 86(40.2%) 55(59.8%) 128(59.8%)	0.996
**Numbers of snacks that you consume**	0–1 2 > 2	133 (43.5%) 107(35.0%) 66(21.6%)	6(66.67%) 61(43.0%) 51(44.7%) 15(36.6%) 2(22.22%) 55(38.7%) 38(33.3%) 12(29.3%) 1(11.1%) 26(18.3%) 25(21.9%) 14(34.1%)	0.299	39(42.4%) 94(43.9%) 27(29.3%) 80(37.4%) 26(28.3%) 40(18.7%)	0.138
**The number of consumption of cereals**	Normal More Less	205(67.0%) 56(18.3%) 45(14.7%)	6(66.67%) 101(71.1%) 69(60.5%) 29(70.7%) 1(11.11%) 23(16.2%) 24(21.1%) 8(19.5%) 2(22.22%) 18(12.7%) 21(18.4%) 4(9.8%)	0.575	65(70.7%) 140(65.4%) 16(17.4%) 40(18.7%) 11(12.0%) 34(15.9%)	0.607
**Consumption of protein during covid-19**	Normal More Less	222(72.5%) 69(22.5%) 15(4.9%)	6(66.67%) 105(73.9%) 82(71.9%) 29(70.7%) 3(33.33%) 31(21.8%) 25(21.9%) 10(24.4%) 0(0.00%) 6(4.2%) 7(6.1%) 2(4.9%)	0.952	61(66.3%) 161(75.2%) 23(25.0%) 46(21.5%) 8(8.7%) 7(3.3%)	0.085
**Consumption of fruit and vegetable**	Normal More Less	157(51.3%) 127(41.5%) 22(7.2%)	6(66.67%) 73(51.4%) 57(50.00%) 21(51.2%) 2(22.22%) 60(42.3%) 48(42.1%) 17(41.5%) 1(11.11%) 9(6.3%) 9(7.9%) 3(7.3%)	0.945	55(59.8%) 102(47.7%) 29(31.5%) 98(45.8%) 8(8.7%) 14(6.5%)	0.067
**Consumption of sweet during covid**	Normal More Less	163(53.3%) 72(23.5%) 71(23.2%)	4(44.44%) 90(63.4%) 54(47.4%) 15(36.6%) 1(11.11%) 23(16.2%) 30(26.3%) 18(43.9%) 4(44.44%) 29(20.4%) 30(26.3%) 8(19.5%)	0.003*	54(58.7%) 109(50.9%) 17(18.5%) 55(25.7%) 21(22.8%) 50(23.4%)	0.339
**Consumption of soft drinks during covid**	Normal More Less	110(35.9%) 54(17.6%) 142(46.4%)	4(44.44%) 47(33.1%) 42(36.8%) 17(41.5%) 3(33.33%) 24(16.9%) 19(16.7%) 8(19.5%) 2(22.22%) 71(50.0%) 53(46.5%) 16(39.0%)	0.632	36(39.1%) 74(34.6%) 13(14.1%) 41(19.2%) 43(46.7%) 99(46.3%)	0.523
**Consumption of natural juices during covid**	Normal More Less	186(60.8%) 64(20.9%) 56(18.3%)	5(55.56%) 87(61.3%) 70(61.4%) 24(58.5%) 0(0.00%) 32(22.5%) 23(20.2%) 9(22.0%) 4(44.44%) 23(16.2%) 21(18.4%) 8(19.5%)	0.430	62(67.4%) 124(57.9%) 16(17.4%) 48(22.4%) 14(15.2%) 42(19.6%)	0.300
**Changes in daily meals during covid**	Normal More Less	209(68.3%) 70(22.9%) 27(8.8%)	6(66.67%) 104(73.2%) 68(59.6%) 31(75.6%) 2(22.22%) 27(19.0%) 32(28.1%) 9(22.0%) 1(11.11%) 11(7.7%) 14(12.3%) 1(2.4%)	0.235	62(67.4%) 147(68.7%) 22(23.9%) 48(22.4%) 8(8.7%) 19(8.9%)	0.961
**Daily meals in car or street**	**0** 1 2 > 2	189(61.8%) 94(30.7%) 13(4.2%) 10(3.3%)	6(66.67%) 96(67.6%) 66(57.9%) 21(51.2%) 3(33.33%) 36(25.48%) 40(35.1%) 15(36.6%) 0(0.00%) 6(4.2%) 6(5.3%) 1(2.4%) 0(0.00%) 4(2.8%) 2(1.8%) 4(9.8%)	0.225	52(56.5%) 137(64.0%) 25(27.2%) 69(32.2%) 7(7.6%) 6(2.8%) 8(8.7%) 2(0.9%)	0.001*
**Daily meals at restaurant**	**0** 1 > 2	236(77.1%) 66(21.6%) 4(1.3%)	8(88.9%) 108(76.1%) 90(78.9%) 30(73.2%) 1(11.1%) 33(23.2%) 22(19.3%) 10(24.4%) 0(0.00%) 1(0.7%) 2(1.8%) 1(2.4%)	0.872	68(73.9%) 168(78.5%) 24(26.1%) 42(19.6%) 0(00.00%) 4(1.9%)	0.208
**Daily meals or snacks while watching TV**	**0** **1** **2** **> 2**	64(20.9%) 132(43.1%) 63(20.6%) 47(15.4%)	2(22.22%) 32(22.5%) 24(21.1%) 6(14.6%) 4(44.44%) 62(43.7%) 49(43.00%) 17(41.5%) 2(22.22%) 25(17.6%) 26(22.8%) 10(24.4%) 1(11.11%) 23(16.2%) 15(13.2%) 8(19.5%)	0.957	24(26.1%) 40(18.7%) 34(37.0%) 98(45.8%) 20(21.7%) 43(20.1%) 14(15.2%) 33(15.4%)	0.402
**Appetite “eating desire “changes during covid − 19**	Normal More Less	165(53.9%) 85(27.8%) 56(18.3%)	6(66.67%) 89(62.7%) 52(45.6%) 18(43.9%) 1(11.11%) 23(16.2%) 41(36.0%) 20(48.8%) 2(22.22%) 30(21.1%) 21(18.4%) 3(7.3%)	0.001*	51(55.4%) 114(53.3%) 25(27.2%) 60(28.0%) 16(17.4%) 40(18.7%)	0.936
**Exercise rate during covid − 19**	Normal More Less No	71(23.2%) 32(10.5%) 93(30.4%) 110(35.9%)	3(33.33%) 40(28.2%) 23(20.2%) 5(12.2%) 1(11.11%) 16(11.3%) 13(11.4%) 2(4.9%) 1(11.11%) 42(29.6%) 35(30.7%) 15(36.6%) 4(44.44%) 44(31.0%) 43(37.7%) 19(46.3%)	0.347	17(18.5%) 54(25.2%) 13(14.1%) 19(8.9%) 32(34.8%) 61(28.5%) 30(32.6%) 80(37.4%)	0.240
**Walking rate in neighborhood during covid − 19**	Normal More Less No	88(28.8%) 18(5.9%) 115(37.6%) 85(27.8%)	3(33.33%) 50(35.2%) 29(25.4%) 6(14.6%) 1(11.11%) 7(4.9%) 8(7.00%) 2(4.9%) 1(11.11%) 48(33.8%) 44(38.6%) 22(53.7%) 4(44.44%) 37(26.1%) 33(28.9%) 11(26.8%)	0.184	34(37.0%) 54(25.2%) 6(6.5%) 12(5.6%) 38(41.3%) 77(36.0%) 14(15.2%) 71(33.2%)	0.011*
**Trail or bicycle use during covid − 19**	Normal More Less No	39(12.7%) 6(2.0%) 21(6.9%) 240(78.4%)	1(11.11%) 21(14.8%) 14(12.3%) 3(7.3%) 0(0.00%) 3(2.1%) 2(1. 8%) 1(2.4%) 0(0.00%) 11(7.7%) 4(3.5%) 6(14.6%) 8(88.89%) 107(75.4%) 94(82.5%) 31(75.6%)	0.480	16(17.4%) 23(10.7%) 3(3.3%) 3(1.4%) 8(8.7%) 13(6.1%) 65(70.7%) 175(81.8%)	0.170
**Practice any sports?**	**Yes** **No**	96(31.4%) 210(68.6%)	4(44.44%) 46(32.4%) 40(35.1%) 6(14.6%) 5(55.56%) 96(67.6%) 74(64.9%) 35(85.4%)	0.077	47(51.1%) 49(22.9%) 45(48.9%) 165(77.1%)	0.001*
**Seeking nutritional advice from nutritional specialist during covid − 19**	Normal More Less No	62(20.3%) 40(13.1%) 27(8.8%) 177(57.8%)	0(0.00%) 32(22.5%) 23(20.2%) 7(17.1%) 2(22.22%) 18(12.7%) 14(12.3%) 6(14.6%) 2(22.22%) 9(6.3%) 11(9.6%) 5(12.2%) 5(55.56%) 83(58.5%) 66(57.9%) 23(56.1%)	0.679	24(26.1%) 38(17.8%) 11(12.0%) 29(13.6%) 7(7.6%) 20(9.3%) 50(54.3%) 127(59.3%)	0.419
**Seeking physical activity advice by physical therapist during covid − 19**	Normal More Less No	55(18.0%) 26(8.5%) 26(8.5%) 199(65.0%)	0(0.00%) 30(21.1%) 19(16.7%) 6(14.6%) 1(11.11%) 13(9.2%) 9(7.9%) 3(7.3%) 3(33.33%) 8(5. 6%) 11(9.6%) 4(9.8%) 5(55.56%) 91(64.1%) 75(65.8%) 28(68.3%)	0.249	18(19.6%) 37(17.3%) 10(10.9%) 16(7.5%) 6(6.5%) 20(9.3%) 58(63.0%) 141(65.9%)	0.628
**Knowledge of obesity health effects**	**Yes** **No**	275(89.9%) 31(10.1%)	7(77.78%) 127(89.4%) 104(91.2%) 37(90.2%) 2(22.22%) 15(10.6%) 10(8.8%) 4(9.8%)	0.634	81(88.0%) 194(90.7%) 11(12.0%) 20(9.3%)	0.488
**Alcohol intake rate during covid-19**	Normal More Less No	6(2.0%) 7(2.3%) 3(1.0%) 290(94.8%)	0(00.00%) 4(2.8%) 1(0.9%) 1(2.4%) 0(00.00%) 1(0.7%) 6(5.3%) 0(00.00%) 0(00.00%) 0(00.00%) 3(2.6%) 0(00.00%) 9(100.0%) 137(96.5%) 104(91.2%) 40(97.6%)	0.128	2(2.2%) 4(1.9%) 2(2.2%) 5(2.3%) 3(3.3%) 0(0.00%) 85(92.4%) 205(95.8%)	0.069
**Cigarette smoking rate during covid − 19**	Normal More Less No	9(2.9%) 3(1%) 2(0.7%) 292(95.4%)	0(00.00%) 4(2.8%) 3(2.6%) 2(4.9%) 0(00.00%) 2(1.4%) 1(0.9%) 0(00.00%) 0(00.00%) 0(00.00%) 1(0.9%) 1(2.4%) 9(100.0%) 136(95.8%) 109(95.6%) 38(92.7%)	0.857	5(5.4%) 4(1.9%) 3(3.3%) 0(0.00%) 2(2.2%) 0(0.00%) 82(89.1%) 210(98.1%)	0.002*
**Behavior changes during covid − 19**	**Yes** **No**	197(64.4%) 109(35.6%)	3(33.33%) 96(67.6%) 66(57.9%) 32(78.0%) 6(66.67%) 46(32.4%) 48(42.1%) 9(22.0%)	0.02*	49(53.3%) 148(69.2%) 43(46.7%) 66(30.8%)	0.008*
**Insomnia rate during covid-19**	Normal More Less No	114(37.3%) 102(33.3%) 35(11.4%) 55(18.0%)	3(33.33%) 53(37.3%) 48(42.1%) 10(24.4%) 1(11.11%) 49(34.5%) 31(27.2%) 21(51.2%) 2(22.22%) 17(12.0%) 12(10.5%) 4(9.8%) 3(33.33%) 23(16.2%) 23(20.2%) 6(14.6%)	0.196	37(40.2%) 77(36.0%) 26(28.3%) 76(35.5%) 12(13.0%) 23(10.7%) 17(18.5%) 38(17.8%)	0.650
**Changing in sleeping hours during covid − 19**	Normal More Less	115(37.6%) 152(49.7%) 39(12.7%)	5(55.56%) 60(42.3%) 40(35.1%) 10(24.4%) 4(44.44%) 69(48.6%) 54(47.4%) 25(61.0%) 0(0.00%) 13(9.2%) 20(17.5%) 6(14.6%)	0.138	36(39.1%) 79(36.9%) 47(51.1%) 105(49.1%) 9(9.8%) 30(14.0%)	0.594
**Rate of multivitamins intake during covid − 19**	Normal More Less No	106(34.6%) 97(31.7%) 17(5.6%) 86(28.1%)	4(44.44%) 56(39.4%) 34(29.8%) 12(29.3%) 2(22.22%) 49(34.5%) 31(27.2%) 15(36.6%) 0(0.00%) 4(2.8%) 10(8.8%) 3(7.3%) 3(33.33%) 33(23.2%) 39(34.2%) 11(26.8%)	0.241	26(28.3%) 80(37.4%) 27(29.3%) 70(32.7%) 8(8.7%) 9(42%) 31(33.7%) 55(25.7%)	0.134

Pearson’s Chi-squared test (for categorical data) and Student’s t-test (for continuous normal data) were used to analyze differences in proportions of the tested variables. (*) significant differences at (*p* < 0.05), BMI: Body mass index

### Participant´s changes in dietary, behavioral and weight changes in relation to body mass index

A substantial variation in weight was seen based on the participants’ BMI during the COVID-19 quarantine (Pearson’s χ2, *p* < 0.001), with 68.3% and 43% of the obese and overweight persons, respectively, reporting a rise in weight. Nonetheless, within the same time frame, 66.7% of slim people and 65.5% of those with a normal weight did not see any changes in their weight. Sweets consumption decreased in 44.4% of underweight participants but increased in 43.9% of obese participants (Pearson’s χ2, *p* = 0.003). Just 7.3% and 18.4% of the obese and overweight participants, respectively, as well as 21.1% of the normal weight and 22.2% of the underweight participants showed a decrease in eating desire (Pearson’s χ2, *p* < 0.001). Only one third (33.3%) of underweight participants reported having behavioral changes, compared to 78% of obese participants, 57.9% of overweight participants, and 67.6% of normal weight participants. Behavioral changes included depressed mood, loss of interest or pleasure, and thoughts of death or suicide attempt (Pearson’s χ2, *p* < 0.05) as shown in Table 2.

### Participant´s changes in dietary, behavioral and weight changes in relation to gender

Gender did not significantly affect changes in food consumption, the quantity of snacks consumed, or daily routines throughout the quarantine. A decrease in soft drink intake was observed in 46.7% of males and 46.3% of females (Pearson’s χ2, *p* = 0.523), whereas consumption of fruits and vegetables increased in 31.5% of males and 45.8% of females (Pearson’s χ2, *p* = 0.067). While the majority of the males and females, 56.5% and 64%, respectively, did not have any daily takeaway meals, 27.2% and 32.2% of the males and females, had one daily takeaway meal in a car or on the street (Pearson’s χ2, *p* < 0.001). With 36% of the participants being female and 41.3% of the participants being male, the neighborhood walks lost their appeal. Apart from 15.2% of men, 33.2% of women did not stroll around the neighborhood at all while under quarantine (Pearson’s χ2, *p* = 0.011). In terms of cigarette smoking rates, 1.9% of females and 5.4% of males continued to smoke as they had done before the quarantine (Pearson’s χ2, *p* = 0.002). There was a discernible difference in the sports practices of the sexes during quarantine; 51.1% of the men participated in sports, while 77.1% of the women did not practice any sport (Pearson’s χ2, *p* < 0.001). Male and female gender differences in behavior were significant, accounting for 53.3% of males and 69.2% of females (Pearson’s χ2, *p* = 0.008) as explored in Table 2.

### Participant´s changes in dietary, behavioral and weight changes in relation to current job

As revealed in Table 3, The Participants current job was categorized as students, employed, retired, and free individuals. Consumption of cereals remained normal in (52.7%) of employed participants, (73.2%) of free participants, (50%) of retired and (73.3%) of students (Pearson’s χ2, *p* = 0.023). Regarding proteins intake, it followed the same pattern as (69.2%) of employed, (80.5%) of free, (50%) of retired and (72.7%) of students-maintained proteins standard intake (Pearson’s χ2, *p* = 0.039). Most of the participants declared that their daily meals remained the same as before Covid-19; (70.3%) of employed, (65.9%) of free, and (68.6%) of students, however all retired participants had more daily meals (Pearson’s χ2, *p* = 0.032). Around (74.7%) of employed participants didn’t practice any sports, in addition to (82.9%) of free participants, (100%) of retired, and (61.6%) of students (Pearson’s χ2, *p* = 0.016). Behavioral changes showed considerable variation between these sectors as just over half of employed ones (51.6%) didn’t suffer from any behavior changes, in opposition to (70.7%) of free individuals had behavior changes (Pearson’s χ2, *p* = 0.002), and (71.5%) of students. Besides, (48.4%) of the employed participants reported no alteration in their sleeping hours, while (53.7%) of the free ones and (56.4%) of the students had more sleeping hours in comparison to the period before pandemic (Pearson’s χ2, *p* = 0.028).

### Participant´s changes in dietary, behavioral and weight changes in relation to living home

The participants’ residences have an enormous effect on their daily schedules and dietary decisions. Just 22.3% of participants who lived with family showed an increasing tendency in terms of sweets consumption, compared to 46.7% of participants who lived alone or with friends (Pearson’s χ2, *p* < 0.05). Approximately 46.7% of individuals who were alone or living with friends reported having more meals per day than they did previously, while 69.8% of participants who were living with family reported no change in daily meal consumption (Pearson’s χ2, *p* < 0.05). About half of the respondents who were alone (40%) said they never had takeout, compared to a higher percentage of those who were living with family (62.9%) who never had takeaway. It was discovered that this difference was statistically significant (*p* = 0.002). 32.6% of participants who were living with relatives increased their multivitamin intake during the quarantine, compared to 46.7% of participants who were living alone or with friends (Pearson’s χ2, *p* < 0.02) as presented in Table 3.

**Table 3:** Participants’ dietary, behavioral, and weight changes related to job, living home, and family obesity history

Questions	Response	Total	Current Job Employed (*n* = 91) Free (*n* = 41) Retired (*n* = 2) Student(*n* = 172)	*P*	Living Home Alone( *n* = 15) w` family (*n* = 291 )	*P*	Family History of Obesity Yes(*n* = 109) NO(*n* = 197)	*P*
**Weight change during covid-19**	Normal Increase Decrease	153 (50.0%) 104(34.0%) 49(16.0%)	43(47.3%) 21(51.2%) 1(50.0%) 88(51.2%) 31(34.1%) 17(41.5%) 1(50.0%) 55(32.0%) 17(18.7%) 3(7.3%) 0(0.00%) 29(16.9%)	0.682	5 (33.3%) 148(50.9%) 7(46.7%) 97(33.3%) 3(20.0%) 46(15.8%)	0.411	45(41.3%) 108(54.8%) 38(34.9%) 66(33.5%) 26(23.9%) 23(11.7%)	0.011*
**Weight change is intended**	Yes No	123(40.2%) 183(59.8%)	39(42.9%) 12(29.3%) 2(100.0%) 70(40.7%) 52(57.1%) 29(70.7%) 0(0.00%) 102(59.3%)	0.151	8(53.3%) 115(39.5%) 7(46.7%) 176(60.5%)	0.287	59(54.1%) 64(32.5%) 50(45.9%) 133(67.5%)	0.001*
**Numbers of snacks that you consume**	0–1 2 > 2	133 (43.5%) 107(35.0%) 66(21.6%)	37(40.7%) 19(46.3%) 0(0.00%) 77(44.8%) 33(36.3%) 12(29.3%) 1(50.0%) 61(35.5%) 21(23.1%) 10(24.4%) 1(50.0%) 34(19.8%)	0.809	3(20.0%) 130(44.7%) 8(53.3%) 99(34.0%) 4(26.7%) 62(21.3%)	0.156	49(45.0%) 84(42.6%) 34(31.2%) 73(37.1%) 26(23.9%) 40(20.3%)	0.553
**The number of consumption of cereals**	Normal More Less	205(67.0%) 56(18.3%) 45(14.7%)	48(52.7%) 30(73.2%) 1(50.0%) 126(73.3%) 26(28.6%) 6(14.6%) 1(50.0%) 23(13.4%) 17(18.7%) 5(12.2%) 0(0.00%) 23(13.4%)	0.023*	8(53.3%) 197(67.7%) 3(20.0%) 53(18.2%) 4(26.7%) 41(14.1%)	0.368	72(66.1%) 133(67.5%) 16(14.7%) 40(20.3%) 21(19.3%) 24(12.2%)	0.163
**Consumption of protein during covid-19**	Normal More Less	222(72.5%) 69(22.5%) 15(4.9%)	63(69.2%) 33(80.5%) 1(50.0%) 125(72.7%) 21(23.1%) 8(19.5%) 0(0.00%) 40(23.3%) 7(7.7%) 0(0.00%) 1(50.0%) 7(4.1%)	0.039*	10(66.7%) 212(72.9%) 4(26.7%) 65(22.3%) 1(6.7%) 14(4.8%)	0.863	76(69.7%) 146(74.1%) 29(26.6%) 40(20.3%) 4(3.7%) 11(5.6%)	0.378
**Consumption of fruit and vegetable**	Normal More Less	157(51.3%) 127(41.5%) 22(7.2%)	54(59.3%) 21(51.2%) 1(50.0%) 81(47.1%) 30(33.0%) 16(39.0%) 0(0.00%) 81(47.1%) 7(7.7%) 4(9.8%) 1(50.0%) 10(5.8%)	0.075	9(60.0%) 148(50.9%) 4(26.7%) 123(42.3%) 2(13.3%) 20(6.9%)	0.387	48(44.0%) 109(55.3%) 55(50.5%) 72(36.5%) 6(5.5%) 16(8.1%)	0.058
**Consumption of sweet during covid**	Normal More Less	163(53.3%) 72(23.5%) 71(23.2%)	51(56.0%) 21(51.2%) 1(50.0%) 90(52.3%) 21(23.1%) 10(24.4%) 1(50.0%) 40(23.3%) 19(20.9%) 10(24.4%) 0(0.00%) 42(24.4%)	0.950	8(53.3%) 155(53.3%) 7(46.7%) 65(22.3%) 0(0.00%) 71(24.4%)	0.027*	56(51.4%) 107(54.3%) 19(17.4%) 53(26.9%) 34(31.2%) 37(18.8%)	0.024*
**Consumption of soft drinks during covid**	Normal More Less	110(35.9%) 54(17.6%) 142(46.4%)	35(38.5%) 19(46.3%) 1(50.0%) 55(32.0%) 12(13.2%) 7(17.1%) 1(50.0%) 34(19.8%) 44(48.4%) 15(36.6%) 0(0.00%) 83(48.3%)	0.331	6(40.0%) 104(35.7%) 3(20.0%) 51(17.5%) 6(40.0%) 136(46.7%)	0.878	37(33.9%) 73(37.1%) 19(17.4%) 35(17.8%) 53(48.6%) 89(45.2%)	0.830
**Consumption of natural juices during covid**	Normal More Less	186(60.8%) 64(20.9%) 56(18.3%)	56(61.5%) 23(56.1%) 0(0.00%) 107(62.2%) 17(18.7%) 8(19.5%) 1(50.0%) 38(22.1%) 18(19.8%) 10(24.4%) 1(50.0%) 27(15.7%)	0.508	9(60.0%) 177(60.8%) 2(13.3%) 62(21.3%) 4(26.7%) 52(17.9%)	0.595	67(61.5%) 119(60.4%) 19(17.4%) 45(22.8%) 23(21.1%) 33(16.8%)	0.423
**Changes in daily meals during covid**	Normal More Less	209(68.3%) 70(22.9%) 27(8.8%)	64(70.3%) 27(65.9%) 0(0.00%) 118(68.6%) 15(16.5%) 13(31.7%) 2(100.0%) 40(23.3%) 12(13.2%) 1(2.4%) 0(0.00%) 14(8.1%)	0.032*	6(40.0%) 203(69.8%) 7(46.7%) 63(21.6%) 2(13.3%) 25(8.6%)	0.047*	76(69.7%) 133(67.5%) 20(18.3%) 50(25.4%) 13(11.9%) 14(7.1%)	0.181
**Daily meals in car or street**	**0** 1 2 > 2	189(61.8%) 94(30.7%) 13(4.2%) 10(3.3%)	50(54.9%) 23(56.1%) 2(100.0%) 114(66.3%) 30(33.0%) 15(36.6%) 0(0.00%) 49(28.5%) 4(4.4%) 2(4.9%) 0(0.00%) 7(4.1%) 7(7.7%) 1(2.4%) 0(0.00%) 2(1.2%)	0.242	6(40.0%) 183(62.9%) 5(33.3%) 89(30.6%) 1(6.7%) 12(4.1%) 3(20.0%) 7(2.4%)	0.002*	65(59.6%) 124(62.9%) 33(30.3%) 61(31.0%) 6(5.5%) 7(3.6%) 5(4.6%) 5(2.5%)	0.645
**Daily meals at restaurant**	**0** 1 > 2	236(77.1%) 66(21.6%) 4(1.3%)	65(71.4%) 32(78.0%) 2(100.0%) 137(79.7%) 25(27.5%) 8(19.5%) 0(0.00%) 33(19.2%) 1(1.1%) 1(2.4%) 0(0.00%) 2(1.2%)	0.732	8(53.3%) 228(78.4%) 7(46.7%) 59(20.3%) 0(00.00%) 4(1.4%)	0.051	77(70.6%) 159(80.7%) 30(27.5%) 36(18.3%) 2(1.8%) 2(1.0%)	0.131
**Daily meals or snacks while watching TV**	**0** **1** **2** **> 2**	64(20.9%) 132(43.1%) 63(20.6%) 47(15.4%)	15(16.5%) 10(24.4%) 0(0.00%) 39(22.7%) 48(52.7%) 14(34.1%) 0(0.00%) 70(40.7%) 19(20.9%) 10(24.4%) 1(50.0%) 33(19.2%) 9(9.9%) 7(17.1%) 1 (50.0%) 30(17.4%)	0.291	2(13.3%) 62(21.3%) 6(40.0%) 126(43.3%) 6(40.0%) 57(19.6%) 1(6.7%) 46(15.8%)	0.248	27(24.8%) 37(18.8%) 42(38.5%) 90(45.7%) 22(20.2%) 41(20.8%) 18(16.5%) 29(14.7%)	0.533
**Appetite “eating desire “changes during covid − 19**	Normal More Less	165(53.9%) 85(27.8%) 56(18.3%)	50(54.9%) 21(51.2%) 0(0.00%) 94(54.7%) 23(25.3%) 14(34.1%) 2(100.0%) 46(26.7%) 18(19.8%) 6(14.6%) 0(0.00%) 32(18.6%)	0.362	7(46.7%) 158(54.3%) 6(40.0%) 79(27.1%) 2(13.3%) 54(18.6%)	0.545	55(50.5%) 110(55.8%) 33(30.3%) 52(26.4%) 21(19.3%) 35(17.8%)	0.656
**Exercise rate during covid − 19**	Normal More Less No	71(23.2%) 32(10.5%) 93(30.4%) 110(35.9%)	24(26.4%) 9(22.0%) 0(0.00%) 38(22.1%) 7(7.7%) 4(9.8%) 0(0.00%) 21(12.2%) 21(23.1%) 15(36.6%) 1(50.0%) 56(32.6%) 39(42.9%) 13(31.7%) 1(50.0%) 57(33.1%)	0.646	6(40.0%) 65(22.3%) 1(6.7%) 31(10.7%) 3(20.0%) 90(30.9%) 5(33.3%) 105(36.1%)	0.436	24(22.0%) 47(23.9%) 14(12.8%) 18(9.1%) 34(31.2%) 59(29.9%) 37(33.9%) 73(37.1%)	0.741
**Walking rate in neighborhood during covid − 19**	Normal More Less No	88(28.8%) 18(5.9%) 115(37.6%) 85(27.8%)	33(36.3%) 14(34.1%) 0(0.00%) 41(23.8%) 7(7.7%) 1(2.4%) 0(0.00%) 10(5.8%) 27(29.7%) 15(36.6%) 0(0.00%) 73(42.4%) 24(26.4%) 11(26.8%) 2(100.0%) 48(27.9%)	0.169	6(40.0%) 82(28.2%) 1(6.7%) 17(5.8%) 6(40.0%) 109(37.5%) 2(13.3%) 83(28.5%)	0.589	33(30.3%) 55(27.9%) 8(7.3%) 10(5.1%) 42(38.5%) 73(37.1%) 26(23.9%) 59(29.9%)	0.631
**Trail or bicycle use during covid − 19**	Normal More Less No	39(12.7%) 6(2.0%) 21(6.9%) 240(78.4%)	10(11.0%) 5(12.2%) 0(0.00%) 24(14.0%) 5(5.5%) 0(0.00%) 0(0.00%) 1(0.6%) 5(5.5%) 4(9.8%) 0(0.00%) 12(7.0%) 71(78.0%) 32(78.0%) 2(100.0%) 135(78.5%)	0.351	2(13.3%) 37(12.7%) 1(6.7%) 5(1.7%) 0(0.00%) 21(7.2%) 12(80.0%) 228(78.4%)	0.412	13(11.9%) 26(13.2%) 3(2.8%) 3(1.5%) 6(5.5%) 15(7.6%) 87(79.8%) 153(77.7%)	0.771
**Practice any sports?**	**Yes** **No**	96(31.4%) 210(68.6%)	23(25.3%) 7(17.1%) 0(0.00%) 66(38.4%) 68(74.7%) 34(82.9%) 2(100.0%) 106(61.6%)	0.016*	7(46.7%) 89(30.6%) 8(53.3%) 202(69.4%)	0.191	38(34.9%) 58(29.4%) 71(65.1%) 139(70.6%)	0.328
**Seeking nutritional advice from nutritional specialist during covid − 19**	Normal More Less No	62(20.3%) 40(13.1%) 27(8.8%) 177(57.8%)	19(20.9%) 12(29.3%) 0(0.00%) 31(18.0%) 9(9.9%) 5(12.2%) 0(0.00%) 26(15.1%) 9(9.9%) 6(14.6%) 0(0.00%) 12(7.0%) 54(59.3%) 18(43.9%) 2(100.0%) 103(59.9%)	0.465	4(26.7%) 58(19.9%) 1(6.7%) 39(13.4%) 1(6.7%) 26(8.9%) 9(60.0%) 168(57.7%)	0.823	21(19.3%) 41(20.8%) 20(18.3%) 20(10.2%) 9(8.3%) 18(9.1%) 59(54.1%) 118(59.9%)	0.245
**Seeking physical activity advice by physical therapist during covid − 19**	Normal More Less No	55(18.0%) 26(8.5%) 26(8.5%) 199(65.0%)	13(14.3%) 11(26.8%) 0(0.00%) 31(18.0%) 10(11.0%) 1 (2.4%) 0(0.00%) 15(8.7%) 7(7.7%) 5(12.2%) 0(0.00%) 14(8.1%) 61(67.0%) 24(58.5%) 2(100.0%) 112(65.1%)	0.629	3(20.0%) 52(17.9%) 1(6.7%) 25(8.6%) 1(6.7%) 25(8.6%) 10(66.7%) 189(64.9%)	0.983	21(19.3%) 34(17.3%) 11(10.1%) 15(7.6%) 12(11.0%) 14(7.1%) 65(59.6%) 134(68.0%)	0.443
**Knowledge of obesity health effects**	**Yes** **No**	275(89.9%) 31(10.1%)	81(89.0%) 38(92.7%) 2(100.0%) 154(89.5%) 10(11.0%) 3(7.3%) 0(0.00%) 18(10.5%)	0.879	12(80.0%) 263(90.4%) 3(20.0%) 28(9.6%)	0.194	106(97.2%) 169(85.8%) 3(2.8%) 28(14.2%)	0.001*
**Alcohol intake rate during covid-19**	Normal More Less No	6(2.0%) 7(2.3%) 3(1.0%) 290(94.8%)	3(3.3%) 0(0.00%) 0(0.00%) 3(1.7%) 0(0.00%) 2(4.9%) 0(0.00%) 5(2.9%) 2(2.2%) 0(0.00%) 0(0.00%) 1(0.6%) 86(94.5%) 39(95.1%) 2(100.0%) 163(94.8%)	0.594	0(0.00%) 6(2.1%) 0(0.00%) 7(2.4%) 0(0.00%) 3(1.0%) 15(100.0%) 275(94.5%)	0.833	2(1.8%) 4(2.0%) 2(1.8%) 5(2.5%) 1(0.9%) 2(1.0%) 104(95.4%) 186(94.4%)	0.981
**Cigarette smoking rate during covid − 19**	Normal More Less No	9(2.9%) 3(1%) 2(0.7%) 292(95.4%)	3(3.3%) 0(0.00%) 0(0.00%) 6(3.5%) 0(0.00%) 0(0.00%) 0(0.00%) 3(1.7%) 2(2.2%) 0(0.00%) 0(0.00%) 0(0.00%) 86(94.5%) 41(100.0%) 2(100.0%) 163(94.8%)	0.470	1(6.7%) 8(2.7%) 1(6.7%) 2(0.7%) 0(0.00%) 2(0.7%) 13(86.7%) 279(95.9%)	0.103	5(4.6%) 4(2.0%) 0(0.00%) 3(1.5%) 1(0.9%) 1(0.5%) 103(94.5%) 189(95.9%)	0.332
**Behavior changes during covid − 19**	**Yes** **No**	197(64.4%) 109(35.6%)	44(48.4%) 29(70.7%) 1(50.0%) 123(71.5%) 47(51.6%) 12(29.3%) 1(50.0%) 49(28.5%)	0.002*	10(66.7%) 187(64.3%) 5(33.3%) 104(35.7%)	0.850	74(67.9%) 123(62.4%) 35(32.1%) 74(37.6%)	0.340
**Insomnia rate during covid-19**	Normal More Less No	114(37.3%) 102(33.3%) 35(11.4%) 55(18.0%)	39(42.9%) 19(46.3%) 0(0.00%) 56(32.6%) 21(23.1%) 14(34.1%) 2(100.0%) 65(37.8%) 15(16.5%) 4(9.8%) 0(0.00%) 16(9.3%) 16(17.6%) 4(9.8%) 0(0.00%) 35(20.3%)	0.078	6(40.0%) 108(37.1%) 4(26.7%) 98(33.7%) 4(26.7%) 31(10.7%) 1(6.7%) 54(18.6%)	0.207	33(30.3%) 81(41.1%) 51(46.8%) 51(25.9%) 9(8.3%) 26(13.2%) 16(14.7%) 39(19.8%)	0.003*
**Changing in sleeping hours during covid − 19**	Normal More Less	115(37.6%) 152(49.7%) 39(12.7%)	44(48.4%) 15(36.6%) 0(0.00%) 56(32.6%) 32(35.24%) 22(53.7%) 1(50.0%) 97(56.4%) 15(16.5%) 4(9.8%) 1(50.0%) 19(11.0%)	0.028*	5(33.3%) 110(37.8%) 6(40.0%) 146(50.2%) 4(26.7%) 35(12.0%)	0.250	29(26.6%) 86(43.7%) 67(61.5%) 85(43.1%) 13(11.9%) 26(13.2%)	0.006*
**Rate of multivitamins intake during covid − 19**	Normal More Less No	106(34.6%) 97(31.7%) 17(5.6%) 86(28.1%)	34(37.4%) 14(34.1%) 1(50.0%) 57(33.1%) 32(35.2%) 13(31.7%) 0(0.00%) 52(30.2%) 7(7.7%) 3(7.3%) 0(0.00%) 7(4.1%) 18(19.8%) 11(26.8%) 1(50.0%) 56(32.6%)	0.623	3(20.0%) 103(35.4%) 2(13.3%) 95(32.6%) 3(20.0%) 14(4.8%) 7(46.7%) 79(27.1%)	0.015*	35(32.1%) 71(36.0%) 43(39.4%) 54(27.4%) 5(4.6%) 12(6.1%) 26(23.9%) 60(30.5%)	0.180

Pearson’s Chi-squared test (for categorical data) and Student’s t-test (for continuous normal data) were used to analyze differences in proportions of the tested variables. (*) significant differences at (*p* < 0.05)

### Participant´s changes in dietary, behavioral and weight changes in relation to family history of obesity

Individuals were categorized based on whether or not there was a family history of obesity. During the quarantine period, the weight of both those without a family history of obesity (54.8%) and those with a family history of obesity (41.3%) remained normal (Pearson’s χ2, *p* < 0.02). Of the participants without a family history of obesity, 67.5% made unintentional weight adjustments, whereas 54.1% of those with a family history of obesity had planned to make weight changes (Pearson’s χ2, *p* < 0.001). In terms of candy consumption, individuals without a family history of obesity exhibited an increase in intake (26.9%), whereas those with a family history of obesity saw a decrease in intake (31.2%) (Pearson’s χ2, *p* = 0.024). Just 2.8% of participants with a family history of obesity were unaware of the negative health impacts of obesity, compared to about 14.2% of those without a family history (Pearson’s χ2, *p* = 0.001). The rate of insomnia also varied; in 41.1% of people without a family history of obesity, it stayed normal, whereas in 46.8% of participants with a family history of obesity, it increased (Pearson’s χ2, *p* < 0.01). Additionally, 43.1% of patients without a family history reported sleeping longer during quarantine than they did prior to COVID-19, while 43.7% of participants with no family history reported no changes in sleeping duration (Pearson’s χ2, *p* < 0.01). On the other side, 61.5% of participants with a family history of obesity had more sleeping hours as displayed in Table 3.

## Discussion

Covid-19 lockdown have greatly affected people’s daily lives. This study represents the first thorough analysis of how rural Egyptians’ behavior changed during the COVID-19 quarantine. The results of the study have illuminated notable shifts in a number of participant behaviors, including weight, sweets consumption, eating urges, and behavior, all of which showed a strong relationship with body mass index (BMI). The study also discovered significant changes in daily meal patterns, such as eating meals on the go or in cars, as well as behavioral and physical activity changes that were found to be gender-dependent. Additionally, the study emphasized the influence of living circumstances, showing notable variations in the amount of sweets ingested, the number of meals eaten each day and the frequency of multivitamin consumption depending on the kind of living arrangement. Furthermore, the research found strong associations between weight, desire to lose weight, consumption of sweets, awareness of the negative health impacts of obesity, rates of insomnia, length of sleep, and family history of obesity. Lastly, the study looked at the effect of employment position and found statistically significant variations in the amount of cereal and protein consumed as well as the frequency of meals each day. These results shed important light on how the rural Egyptian populace responded to the COVID-19 quarantine and the different variables affecting their daily routines during this difficult period.

About half of the individuals in this study (50%) reported no changes in weight during the quarantine period. This is consistent with another study on the Danish population, which found the same percentage, and also with another study on British population in which most individuals (76.4%) remained in the same BMI category post-lockdown, as opposed to the Italian and American studies, which found that 59.7% and 58% of participants had gained weight, respectively [11–14]. This observation may be explained by the distinctive features of the Egyptian food and lifestyle. Additionally, the study found that, in comparison to before the quarantine, 51.3% of individuals continued to consume fruits and vegetables at their usual levels, while 41.5% of participants increased their intake of these foods. While these findings differ from other studies, they are consistent with some of them [11, 15–19]. This increase in fruits and vegetables consumption can be explained by the fact that Egypt has a more varied climate than other countries, which makes a wide range of fruits and vegetables both affordable and readily available throughout the year, particularly in rural areas of Egypt that rely economically on farming and agriculture. Moreover, the disrupted access to fresh groceries during lockdown and the reduced trades between rural and urban regions as reported by The Food and Agriculture Organization (FAO) decreased the possibility of selling fruits and vegetables to urban centers [20]. This situation coupled with the short shelf life of most fruits and vegetables forced farmers to rely more on them in their dietary habits. Furthermore, the increased focus on boosting immunity has shifted the dietary preferences towards eating more fruits and vegetables to overcome the virus’s impact. Another factor to consider is education, most of our participants held a university degree or higher postgraduate education, and our findings indicate that individuals with more than secondary education are likely to maintain healthier eating habits, and consequently consume more fruits and vegetables during the lockdown. This aligns with the previous finding of Robinson et al., which stated that the behavior of higher education level showed a decreased overeating or unhealthy diets than lower education level in UK [21]. Egypt is an agricultural country; thus, home cooking is highly valued. This gives people more control over the nutritional value of their food and can include more fruits and vegetables in their meals than they can at restaurants. Due to the potential of having access to stores, some studies conducted during the pandemic period found a decrease in the purchase of fresh foodstuffs [17, 19, 22, 23]. Fruits and vegetables can help you maintain a healthy body weight when eaten in moderation. According to Shyam et al., individuals in the control group saw weight loss as a result of changing their eating habits. For instance, by adhering to a MedDiet more closely which align with another study [24, 25]. It’s also possible that those who either lost weight or kept it off participated in cultural practices like housework and prayer rituals, which supported their level of physical activity, even if this was not scientifically proven. Furthermore, these behaviors probably affected weight maintenance because the quarantine period fell around Ramadan, when the majority of Egyptians fast for the whole month and observe extra prayers.

Furthermore, this data showed a significant correlation between those with a higher BMI (being obese and overweight) and higher weight gain which is against an English study showed the proportion of obese who moved down at least one BMI category was greater than the proportion who moved up at least one category [13]. On the other hand, people who are underweight or average weight typically maintain their weight, which is in line with the results of other studies [13, 17, 26–29]. Because people with higher BMIs frequently have metabolic adaptations that encourage weight retention and increase, metabolic variables can be attributed to these findings. Changes in hormones that control appetite, a decrease in metabolic rates, or an enhanced perception of taste that heightens the desire for sweet meals are a few examples of these adaptations. The survey also revealed eating habits in relation to BMI, providing further insight into these results. Specifically, 43.9% of individuals who were obese reported consuming more sweets that line with some studies [16, 22, 30]. Compared to only 11.1% of participants who were underweight, which is consistent with other research’ findings [18, 30]. Some studies revealed that there was no association between BMI and eating a balanced diet, but there was a correlation between a higher BMI and consuming more junk food, including dressings, snacks, sweetened beverages, and candies [15, 16, 31–33]. During the COVID-19 self-quarantine, there was an 82% rise in unhealthy food in the home, according to a UK article [29]. It is consistent with other studies that only 11.1% of the skinny participants increased their intake of sweets. About 20% of participants in an Indian survey reported consuming less fast food, fried food, junk food, sweets, and chocolates [34]. The brain’s reward part is often activated by sweet meals, especially in people with higher body mass indices. The extended time of confinement during the quarantine probably exacerbated these yearning reactions. Furthermore, according to the study, eating cravings increased in 48.8% of obese individuals and 36% of overweight participants. This finding may imply that emotional eating occurs when people are stressed out or bored, which results in mindless calorie consumption which is align with one study [35]. Because they spend a lot of time at home under quarantine and frequently have full access to food, these groups are generally known to exhibit more problematic eating behaviors, such as eating when not hungry and overeating frequently [36]. In addition to “mindless eating,” which is eating emotionally in reaction to stress or boredom and increasing calorie intake, certain studies support this theory [15, 17, 28, 35, 37–39]. Furthermore, stress in particular can cause hyperphagia, binge eating, and a change in the kinds of food consumed [40–42]. Nonetheless, 62.7% of people with normal weight and 66.7% of those with underweight had normal eating impulses. Additionally, like in previous studies, 78% of people who were obese, 67.6% of participants who were normal weight, and 57.9% of participants who were overweight experienced behavioral abnormalities such depression, loss of interest, or thoughts of death [27, 37, 43, 44]. High levels of depression have been linked to poor dietary habits, increased consumption of saturated fat, energy-dense foods, and salty foods [45, 46]. In a stressful situation, women who tended to act impulsively were more likely to consume more candy [47]. Anxious people ate snacks 2.45 times more frequently [48]. In contrast, 66.7% of underweight participants denied experiencing these behavioral changes, as they were more capable of engaging in physical exercises (11.1%) or at least maintaining their regular exercise routines (33.3%), which can have protective effects against mood disorders.

Daily routines and eating are greatly influenced by the distinct cultural and social characteristics of Egyptians, especially when it comes to housing arrangements. According to the survey, those who live alone or with friends eat differently from people who live with their family. It was discovered, specifically, that 46.7% of participants who lived alone or with friends consumed more sweets because they had less control over what they ate. This result is in line with earlier studies conducted during the same time period on the food environment [49]. In contrast, only 22.3% of participants living with family showed a similar trend. Zurita et al. revealed that Living in the family home has been associated with a higher quality of diet [50]. When it comes to making decisions, elder generations in Egyptian households usually have a significant influence. They usually prioritize eating balanced meals and healthier options, especially in emergency situations like quarantines and this align with some studies [15, 51]. Other earlier research revealed some encouraging changes, including a decline in the consumption of processed meat and carbonated or sugary beverages and an increase in the consumption of fresh produce, seafood, legumes, and white meat [15, 16]. Additionally, family support and economic assistance play a role in promoting healthier choices and reducing the appeal of sweets. The study also indicated that the number of daily meals was impacted by living arrangements, with 46.7% of participants living alone or with friends having more daily meals than before. This increased flexibility and independence allowed them to control the timing and frequency of their meals without being restricted to scheduled shared family meals. Furthermore, the results showed that individuals living alone or with friends relied more on takeaway meals, which may be attributed to limited cooking skills or busy schedules. Conversely, 69.8% of those living with family reported no changes in their daily meals, and 62.9% completely stopped consuming takeaway meals during the quarantine period as shown in one study [51] while the same study showed opposite direction regarding changes in daily meals. Another notable finding was that increased intake of multivitamins was more prevalent among individuals living with their families (32.6%). Family gatherings often foster a supportive environment that encourages each other to prioritize health and boost the immune system, thereby alleviating feelings of isolation and depression. These results confirm earlier research demonstrating that family/shared meal routines foster emotional well-being because of the opportunities they offer for interpersonal connections, safety and predictability, and communication and sharing of ideas and feelings [52–57].

The participants’ current jobs have had a significant influence on their dietary and daily habits, particularly among those who are employed or retired which is in contrary with another study [30]. The highest increase in cereal consumption was observed among these groups. Cereals are a preferred choice for these participants because they have a long shelf life and can be stocked up during quarantine periods. Additionally, cereals are convenient options for breakfast and snacks, offering high nutritional value. As Price, shelf life, flavor, nutritional value, and hygienic practices are crucial factors to consider while making food purchases [58, 59]. On the other hand, unemployed participants and students, who had more available time due to school and university closures, had the opportunity to explore different food options. According to Zuluğ et al., 80% of the study subjects consented to try several recipes for quarantine [59].

The consumption of proteins followed a similar trend as cereals, with an increase among employed and retired participants. This may be attributed to the financial pressures faced by students and unemployed participants during the quarantine period. Protein-rich foods tend to be more expensive compared to other types of food, making them less affordable for those on limited budgets. Students’ additional free time allowed them to participate in sports, and 38.4% of them did so throughout the quarantine, which is in line with other research but not entirely consistent with others [11, 12, 17]. numerous additional research that demonstrated an increase in sitting or screen time and a reduction in physical activity at all levels [12, 14–16, 23, 37, 44, 46, 48]. Large datasets from France, Spain, and Australia demonstrated that between 43 and 61% of people reduced their physical activity levels during the COVID-19 self-quarantine [17, 23, 60]. According to other studies, a consistent finding was that 40–50% of participants had reduced physical activity [30, 61–64]. While a small number of research revealed the contrary, demonstrating an increase in physical activity during the COVID-19 crisis [16, 65]. These variations might be caused by various government policies regarding restrictions on movement during this time. Despite the limited free time employed participants had during quarantine, it resulted in some drawbacks such as the inability to engage in sports activities. However, it also had certain advantages, including mitigating behavioral changes like depressed mood, loss of interest, or even suicide attempts. Being employed provided these individuals with a sufficient amount of social interaction, even if it was through online conferences or collaboration tools. This social interaction played a crucial role in alleviating the negative effects of isolation and fostering a sense of connection. Moreover, their financial stability and sense of purpose were vital factors in maintaining their mental stability. It has previously been proposed that getting employment predicts having greater health literacy and that being without a profession is linked to not knowing enough about COVID-19 [66–68]. These aspects provided a sense of security and contributed to their overall well-being during the challenging period of quarantine.

Nearly half or more of the participants who were students, unemployed, and retired slept more throughout the quarantine. It was evident, nonetheless, that people who were employed saw the least increase in sleeping hours (35.2%). Stress at work is the cause for this, especially for those who had to make the abrupt switch to remote work. The blurring of the lines separating work and personal life added to the stress and made finding a good work-life balance difficult. All individuals saw an increase in their daily screen use, which can disrupt sleep cycles, particularly if it is done right before bed. Screen blue light can interfere with melatonin release and impair the quality of sleep.

It is essential to note the changes observed in individuals with a family history of obesity, as they are more susceptible to genetic factors that contribute to weight fluctuations [69]. Remarkably, the proportion of participants who gained weight regardless of whether there was a family history of obesity was the same. Despite their apparent propensity for gaining weight, it was interesting to discover that the percentage of people who lost weight was twice as high among those who had a family history of obesity. Furthermore, it was noteworthy that more than half (54.1%) of the weight change observed in participants with a family history of obesity was intentional. This intentionality can be attributed to the findings of our survey, which revealed that nearly all (97.2%) individuals with a family history of obesity had a good understanding of the health effects and consequences of obesity. This heightened awareness of the personal risks they face, along with their genetic predisposition to weight gain, compelled them to take proactive measures to regain control over their bodies. They recognized the existing dangers posed by both their genetic factors and the ongoing pandemic, motivating them to make conscious changes in their daily habits. Our research agrees with some earlier studies that found the majority of participants had sufficient to excellent knowledge and practice about COVID-19 [67, 68]. Our results conflict with those of a study on the Egyptian community in Alexandria that revealed that participants’ levels of health literacy related to COVID-19 are insufficient, as well as another study that reported that most participants had low health literacy [70, 71].

A significant shift in eating patterns was observed in those with a family history of obesity: 31.2% of them reported consuming fewer sweets, compared to only 18.8% of those without such a history. This suggests a conscious attempt to limit their consumption of sugar-filled foods and drinks, which are linked to weight gain. Familial histories of obesity are noteworthy since they are associated with greater incidences of insomnia (46.7%) and longer sleep duration (61.5%). Sleep patterns have been shown to change throughout the COVID-19 self-quarantine; some data indicate that sleep hours have increased [16, 19]. There are several reasons for these seemingly incongruous results. The greater prevalence of insomnia could be attributed to an increase in sleep disruptions and challenges in achieving restorative sleep. Even though people slept for longer periods of time, there have been reports of poor quality and disturbed sleep during the COVID-19 self-quarantine [16, 19, 72]. Additionally, Self-reporting bias may also exist because people who have a genetic predisposition to obesity may be more aware of their sleeping habits and thus report higher rates of insomnia, even when their real sleep quality is comparable to that of people without a family history of obesity.

Our study included some limitations which should be recognized. One of them is the oversampling of specific groups over others. For instance, (70%) of the participants were women, and (73%) participant’s location was in Kafrelsheikh. further studies including more governorates with larger number of participants may be required in the future. In addition, data from rural Egyptian governorate should be compared by other countries in Middle East to explore the real difference in behavior in during Covid-19 lockdown. Also, the absence of urban comparison would be a limitation. Therefore, conducting similar studies is essential to serve as comparable resources for further studies done in different demographics to attain more tailored strategies for different group needs during potential future pandemics.

## Conclusion

Positive changes were observed in Egyptians’ eating habits during the Covid-19 quarantine; nonetheless, daily routines were interrupted by psychological oscillations and a considerable drop-in physical activity. Given that people with a family history of obesity had better health, it was imperative to raise awareness of the implications of obesity. It is important to provide guidance on how to keep activity levels, energy levels balanced, and mood steady. The study offers recommendations for upcoming quarantine circumstances.

## Data Availability

The datasets used and/or analyzed during the current study are available from the corresponding author on reasonable request.

## Abbreviations

COVID: 19 Coronavirus disease
BMI: Body mass index
WHO: World Health Organization
MedDiet: Mediterranean diet
MEDAS: Mediterranean Diet Adherence Screener
